# Efficiency Analysis of Item Response Theory Kernel Equating for Mixed-Format Tests

**DOI:** 10.1177/01466216231209757

**Published:** 2023-10-19

**Authors:** Joakim Wallmark, Maria Josefsson, Marie Wiberg

**Affiliations:** 1 8075 Department of Statistics, USBE, Umeå University, Sweden

**Keywords:** kernel equating, presmoothing, item response theory, log-linear models, simulation

## Abstract

This study aims to evaluate the performance of Item Response Theory (IRT) kernel equating in the context of mixed-format tests by comparing it to IRT observed score equating and kernel equating with log-linear presmoothing. Comparisons were made through both simulations and real data applications, under both equivalent groups (EG) and non-equivalent groups with anchor test (NEAT) sampling designs. To prevent bias towards IRT methods, data were simulated with and without the use of IRT models. The results suggest that the difference between IRT kernel equating and IRT observed score equating is minimal, both in terms of the equated scores and their standard errors. The application of IRT models for presmoothing yielded smaller standard error of equating than the log-linear presmoothing approach. When test data were generated using IRT models, IRT-based methods proved less biased than log-linear kernel equating. However, when data were simulated without IRT models, log-linear kernel equating showed less bias. Overall, IRT kernel equating shows great promise when equating mixed-format tests.

## Introduction

Multiple forms of the same test are commonly administered in large-scale or high-stakes testing programs to ensure adequate testing security. As a result, test scores from multiple forms need to be compared for the purpose of, for example, university program admission. To be able to directly compare test scores from two or more test forms, one must place the scores from each form onto a common scale. This is typically done using a statistical process referred to as *test score equating* (González & Wiberg, 2017; Kolen & Brennan, 2014).

There might be different types of test items in the equated test forms, and the best-performing equating method may depend on the equated forms. Test forms may contain only dichotomously scored items like multiple-choice items, only polytomously scored items which are typically constructed response items or a mixture of the two types. Test forms that contain a mixture of these scoring types are commonly referred to as mixed-format tests (Ercikan et al., 1998; Kim et al., 2008, 2010a, 2010b; Kolen & Lee, 2014). By using a combination of different item formats, tests can be designed to measure a broader set of skills than tests using a single format. Some examples of mixed-format tests include the National Assessment of Educational Progress, the Advanced Placement Program, SAT Reasoning Test, and national tests in mathematics in Sweden.

Although traditional (e.g., Kolen & Brennan, 2014) and item response theory (IRT) equating procedures (Lord, 1980) have been discussed thoroughly in the literature, kernel equating (von Davier et al., 2004) has seen an increase in popularity in the past decades and is, for example, used alongside other equating methods to equate the Swedish scholastic aptitude test (SweSAT). In kernel equating, the test score distributions are typically *presmoothed* before conducting the actual equating in order to remove irregularities in the data due to sampling. In most research, log-linear models have been used to presmooth the test score distributions. We will refer to this method as log-linear kernel equating (LLKE). LLKE has been extensively studied and proven to work well in several different contexts (e.g., von Davier et al., 2004, 2006; Mao et al., 2006; Moses et al., 2007; Liu & Low, 2008).

Andersson and Wiberg (2017) proposed an alternative to log-linear models: the use of dichotomous IRT models for presmoothing. This approach demonstrated promising outcomes when measured against log-linear presmoothing, both in reducing bias and standard errors. This approach can be seen as a fusion of item response theory observed score equating (IRTOSE, Lord, 1980) and LLKE, as it applies IRT models for presmoothing and kernel smoothing for the actual equating. This method, which we denote as item response theory kernel equating (IRTKE), was later extended by Andersson (2016) to be applicable to polytomously scored items using polytomous IRT models. More recently, Wiberg and González (2021) investigated the influence of item discrimination, sample size, and proportions of dichotomous items through simulations in the equivalent groups (EG) design for IRTKE. However, the potential application of IRTKE to mixed-format test forms and comparisons with other IRT-based methods, such as IRTOSE, remain unexplored in previous studies.

The purpose of this research is to assess the efficiency of IRTKE in equating mixed-format tests, under both the EG and NEAT designs. We expand on previous research on IRTKE by comparing it with both LLKE and IRTOSE. Two empirical mixed-format equating examples are presented with real test data from two test forms of the Swedish national test in mathematics, using an EG design, as well as two test forms from the SweSAT, using a non-equivalent groups with anchor test (NEAT) design. In addition, a simulation study using both the EG and NEAT designs is included in order to evaluate the equating transformation performance when different conditions are varied; including the ability of the test taker populations, test form difficulty, and the proportions of dichotomously and polytomously scored items in the test forms.

In the next section, a description of kernel equating together with IRT models for presmoothing is provided. This is followed by the empirical examples. Subsequently, there is an overview of the simulation study, along with the results derived from these simulations. To conclude, the final section offers a discussion on the results obtained, coupled with several suggestions for future research.

## Kernel Equating

Consider a test situation where test form X is administered to a sample from population *P* and test form Y is administered to a sample from population *Q*. Under the EG design, the populations *P* and *Q* are assumed to be equal. Under the NEAT design, the populations are not assumed to be equal and instead an anchor test form, A, containing a set of common items is administered to both samples in order to adjust for population differences when performing the equating. Test scores are considered random variables, represented as *X*, *Y* and *A* for each respective test form X, Y and A. Let the discrete cumulative distribution functions (cdfs) for *X* and *Y* be denoted by *F*_*X*_(*x*) and *F*_*Y*_(*y*). Let *F*_*AP*_(*a*) be the cdf for *A* in population *P*, and *F*_*AQ*_(*a*) be the cdf for *A* in population *Q*. For continuous cdfs, the equipercentile equating transformation is defined $\varphi \left(x\right)=F_{Y}^{-1}\left(F_{X}\left(x\right)\right)$ . As the test score cdfs are typically discrete, kernel equating (von Davier et al., 2004) estimates *φ*(*x*) using continuous approximations of the discrete test score cdfs through kernel smoothing. Although different kernels can be used, the most common choice is to use a Gaussian kernel (von Davier et al., 2004; Mao et al., 2006; Moses et al., 2007; von Davier et al., 2006), which we used for all equatings in this study. When a Gaussian kernel is used, the continuous approximation of *F*_*X*_(*x*) is given by

(1) $$F_{h_{X}}\left(x\right)=\overset{K}{\underset{j=0}{\sum }}r_{j}\Phi \left(\frac{x-u_{X}x_{j}-\left(1-u_{X}\right)\mu_{X}}{u_{X}h_{X}}\right),$$

where *K* is the maximum score on form X, *μ*_*X*_ is the mean of the X scores, *x*_*j*_ is the *j*th score value, *r*_*j*_ is the probability for the *j*th score value, Φ(⋅) denotes the standard normal distribution function, *h*_*X*_ is the bandwidth, and $u_{X}=\sqrt{\frac{\sigma_{X}^{2}}{\sigma_{X}^{2}+h_{X}^{2}}}$ where $\sigma_{X}^{2}$ is the variance *X*. The corresponding approximations of *F*_*Y*_(*y*), *F*_*AP*_(*a*) and *F*_*AQ*_(*a*) are defined analogously and are denoted by $F_{h_{Y}}\left(y\right)$ , $F_{h_{AP}}\left(a\right)$ and $F_{h_{AQ}}\left(a\right)$ . The bandwidth parameter *h*_*X*_ determines the smoothness of $F_{h_{X}}\left(x\right)$ and can be selected using several different methods. As the bandwidth selection method has little effect on the equated values (Wallin et al., 2021), we chose to use the most common method which is to minimize the penalty function

(2) $$\text{PEN}\left(h_{X}\right)=\overset{K}{\underset{j=0}{\sum }}{\left[r_{j}-\frac{d}{dx}F_{h_{X}}\left(x_{j}\right)\right]}^{2}.$$

Under the NEAT design, two competing equating methods exist under the kernel equating framework: chained equating (CE) and post-stratification equating (PSE) (von Davier et al., 2004). Under the EG design and for the NEAT PSE method, the cdfs $F_{h_{X}}\left(x\right)$ and $F_{h_{Y}}\left(y\right)$ are computed for a target population *T,* from which the estimator for the equating transformation is derived $\hat{\varphi }\left(x\right)=F_{h_{Y}}^{-1}\left(F_{h_{X}}\left(x\right)\right)$ . Under the EG design, *T* = *P* = *Q* as the test taker populations are assumed to be equivalent. For NEAT PSE, *T* is defined as the synthetic population *T* = *wP* + (1 − *w*)*Q* where 0 ≤ *w* ≤ 1 is the weight given to *P*. When the NEAT CE method is used, *X* is transformed into *Y* in two-steps. First, *X* is equated to *A* in population *P* and then *A* is equated to *Y* in population *Q* through the equation $\hat{\varphi }\left(x\right)=F_{h_{Y}}^{-1}\left(F_{h_{AQ}}\left(F_{h_{AP}}^{-1}\left(F_{h_{X}}\left(x\right)\right)\right)\right)$ .

Before computing the aforementioned continuous cdfs, *presmoothing* of the univariate (under the EG design) or bivariate (under the NEAT design) score distributions is typically performed to reduce the effect of sampling variation. The use of IRT models for presmoothing is described in the subsequent section. For details on log-linear models, the reader is referred to von Davier et al. (2004).

### IRT Models for Presmoothing

In IRTKE, IRT models are used to presmooth the data. A commonly used model for polytomously scored items is the generalized partial credit (GPC) model (Muraki, 1992). Assuming *M*_*i*_ response categories for item *i*, the GPC model is defined as

(3) $$P_{im}\left(\theta \right)=\left\{ \begin{array}{ll} \frac{1}{1+\sum_{g=1}^{M_{i}-1}\left(\exp\sum_{t=1}^{g}\left[a_{i}\left(\theta -b_{it}\right)\right]\right)}, & \text{if}\;m=1\\ \frac{\exp\left(\overset{m-1}{\underset{t=1}{\sum }}\left[a_{i}\left(\theta -b_{it}\right)\right]\right)}{1+\sum_{g=1}^{M_{i}-1}\left(\exp\sum_{t=1}^{g}\left[a_{i}\left(\theta -b_{it}\right)\right]\right)}, & \text{otherwise} \end{array}\right.$$

where *P*_*im*_(*θ*) is the probability of a test taker with ability *θ* responding in response category *m* on item *i*, *a*_*i*_ is the so-called item discrimination parameter and *b*_*it*_ is the item category difficulty parameter. Note that for dichotomous items (*M*_*i*_ = 2), the GPC model is equivalent to the two parameter logistic model, defined as $P_{i}\left(\theta \right)=(1+\exp{\left(-a_{i}\left(\theta -b_{i}\right)\right)}^{-1}$ , where *b*_*i*_ is the only item difficulty parameter. The underlying assumptions of these models are that the latent variable *θ* is unidimensional and that the responses to each item are conditionally independent given *θ*. After the item probabilities have been modelled using IRT, the probability for each total score conditional on *θ* can be computed using the algorithm described by Thissen et al. (1995). The marginal total score probabilities, *r*_*j*_ in Equation (1), can then be obtained by integrating over *θ*.

## Empirical Examples

To compare the performance of IRTKE, IRTOSE, and LLKE in a practical setting, different datasets were equated under the EG and NEAT designs. The empirical study serves to show how each method functions in a real equating setting, and to obtain a realistic data-generating process for the simulation study. Under the EG design, the 2019 form (X_Math_) of a national test in mathematics was equated to the 2018 form (Y_Math_). The test is given to Swedish high school students taking the mathematics 3c course, a mandatory course for students taking the natural sciences and technology programs. The test has a large impact on the course grade for a student and consists of a mixture of free response items, some requiring short answers and some requiring more in depth step-by-step solutions. Summary statistics for X_Math_ and Y_Math_ are displayed in Table 1. Both X_Math_ and Y_Math_ had 9 dichotomous items and 12 items with three response categories. X_Math_ had three items with four response categories and four items with five response categories, while Y_Math_ had four four-category items and three five-category items, resulting in a one point lower total score on Y_Math_. Strictly speaking, equating is conducted between two forms constructed using the same statistical specifications. Despite the total score differences between the forms, the need to compare test scores from different administrations is still there. Therefore, as the test taker populations are similar in age with similar educational background, the forms were equated under the EG design.

**Table 1.** Summary Statistics for Each Test Form.

Statistic	EG		NEAT			
	X_Math_	Y_Math_	X_SAT_	Y_SAT_	A_SAT_	A_SAT_
Year	2019	2018	2014	2013	2014	2013
Sample size	1401	1008	2859	2469	2859	2469
Number of items	28	28	60	60	30	30
Dichotomous items	9	9	50	50	25	25
Polytomous items	19	19	10	10	5	5
Total score	58	57	80	80	40	40
Mean	25.54	25.72	40.12	40.94	17.56	17.71
Standard deviation	12.62	12.12	12.88	13.34	7.06	7.13
Skewness	.15	.30	.35	.32	.56	0.6
Kurtosis	2.34	2.45	2.49	2.46	2.77	2.78
Correlation with A	—	—	.85	.86	—	—

Under the NEAT design, the 2014 form (X_SAT_) of the SweSAT was equated to the 2013 form (Y_SAT_) with a set of common items (A_SAT_). The SweSAT is used in the higher education application process in Sweden and opportunities to take the test are given twice a year. The test comprises both a verbal and a quantitative part. In this study, only the verbal part was considered. The verbal part consists of three different types of multiple-choice items. The first type is sentence completion, where sentences with missing parts are presented to the test taker and the test taker is asked to select the response alternatives which “fills in the blank.” In the second type, a word is presented and the test taker is asked to select the option corresponding to the meaning of the word. In the third type, the test taker answers several multiple-choice questions related to a text in order to assess reading comprehension. When conducting IRTKE and IRTOSE, the scores on the multiple-choice items of the third type corresponding to the same text were added together and treated as polytomous, as these items cannot be assumed to be independent given test taker ability. Summary statistics for each SweSAT form are displayed in the NEAT columns in Table 1. The polytomous items in test forms X_SAT_ and Y_SAT_ consisted of six items with three response categories, two items with five categories, and two items with six. The anchor test, A_SAT_, had three items with three categories, one item with four and one with five. The summary statistics for the anchor test were almost the same for both the 2013 and 2014 test taker groups, indicating that the test taker populations were similar in ability.

The R programming language (R Core Team, 2021) and the R package kequate (Andersson et al., 2013) were used to conduct LLKE and IRTKE. IRTOSE was performed using our own implementation together with the mirt and equate R packages. For IRTKE and IRTOSE, GPC models were used for presmoothing under both the EG and NEAT designs. Under the NEAT design, IRTKE with PSE and IRTOSE with PSE require the coefficients from each IRT model to be aligned on a common scale through a linking method. The mean-mean method (Kolen & Brennan, 2014) was used for this procedure because it is the only method for polytomously scored items implemented in the kequate package. It is also used in practice when equating the SweSAT. Consequently, it is important to highlight that our results and discussion regarding IRTKE PSE and IRTOSE PSE will exclusively focus on their application in the context of the mean-mean linking method. For LLKE, polynomial log-linear models were used to model the score frequencies for each test form. Under the EG design, order four polynomials were chosen for both test forms based on the Akaike information criterion (AIC). Under the NEAT design, the log-linear models were chosen based on the Bayesian information criterion (BIC), which has been shown to be more efficient than AIC for bivariate smoothing (Moses & Holland, 2010). The bandwidths for each equating method under both designs were selected based on minimization of Equation (2) and are shown in Appendix A.

**Figure 1.**
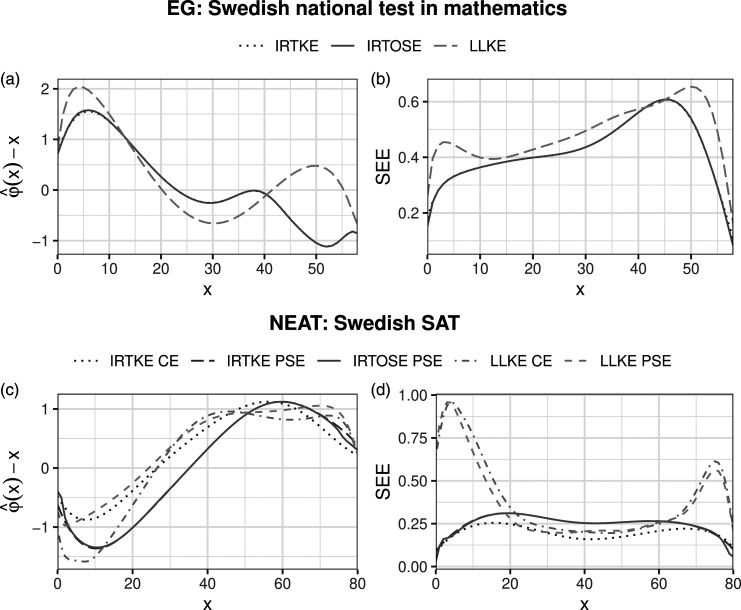
The estimated equating transformations are shown in plots (a) and (c), where the X score is subtracted from the estimated equating transformation for each method. Plots (b) and (d) show the estimated SEEs for each X score.

In order to visualize the differences between LLKE and IRTKE, Figure 1(a) and (c)) show the contrasts between the estimated equating transformations $\hat{\varphi }\left(x\right)$ and their corresponding X_Math_/X_SAT_ scores. A positive difference indicates a higher score on Y_Math_/Y_SAT_ for a certain X_Math_/X_SAT_ score. Recall that the total score on Y_Math_ was one score point lower than on X_Math_ when interpreting Figure 1(a), thus, a difference of zero does not imply equal test form difficulty.

The standard error of equating (SEE) is defined as $\text{SEE}\left(x\right)=\sqrt{\text{Var}\left(\hat{\varphi }\left(x\right)\right)}$ . The estimated SEEs for each equating method are shown in Figure 1(b) and (d). For fair comparisons, these were computed using bootstrap, although analytical derivations also exist for the kernel equating methods, see von Davier et al. (2004) and Andersson (2016) for details.

Under the EG design, the equating functions and the SEEs resulting from IRTKE and IRTOSE are almost indistinguishable. The same is true under the NEAT design when comparing IRTKE PSE against IRTOSE PSE, as they use the same presmoothing method. The effect on the equating transformation from choosing a different presmoothing method (IRT or log-linear) is much larger than the difference between different smoothing methods (kernel or equipercentile).

For the national mathematics test, 1a), all methods suggest that each X_Math_ score corresponds to a marginally higher Y_Math_ score on the lower part of the X_Math_ score scale. The largest differences between IRTKE and LLKE are found for higher scores on X_Math_, reaching magnitudes higher than one score point for scores between 47 and 56. The average value of the equating transformation obtained using IRTKE was .23 points below the average obtained using LLKE. Under the NEAT design (see Figure 1(c)), the equated scores are within 2 points of their corresponding scores on X_SAT_ over the whole score scale for all equating methods. The equating transformations have similar shapes and the largest differences are found at the lower end of the X_SAT_ score scale. All methods suggest that a score over 36 on X_SAT_ equates to a marginally higher score on Y_SAT_.

Under the EG design, the SEEs for IRTKE were marginally lower for most X_Math_ scores compared to the LLKE SEEs, the exceptions being scores in the range 38–49. The average SEE was .61 for IRTKE and .67 for LLKE. Under the NEAT design, the average SEEs were .40, .45, .57, and .52 for IRTKE CE, IRTKE PSE, LLKE CE, and LLKE PSE, respectively. Using IRT models for presmoothing resulted in slightly larger SEEs compared to using log-linear models in the middle score range. However, in the upper and lower ends of the score scales, IRTKE and IRTOSE performed much better than LLKE in terms of SEE.

## Simulation Study

The purpose of the simulation study was to evaluate the performance of IRTKE in comparison with IRTOSE and LLKE for mixed-format tests under the EG and NEAT designs. The R programming language was used for all simulations. The procedures for bandwidth selection, selection of log-linear models, and IRTKE PSE/IRTOSE PSE parameter linking described in the ‘Empirical study' section were also used for the simulations. The simulation code can be obtained at https://github.com/joakimwallmark/kernel-mixed.

A common approach for generating test data for simulations has been through the use of IRT models (Andersson, 2016; Uysal & Kilmen, 2016; Wang & Kolen, 2014). This approach produces realistic looking datasets where it is easy to adjust test taker ability and change item difficulty. However, one drawback is that it might give an advantage to IRT-based equating methods such as IRTKE and IRTOSE when compared to LLKE. Different ways of circumventing this problem have been explored in the literature (e.g., Kim et al., 2008; Leôncio et al., 2022; Leôncio & Wiberg, 2018; Wang et al., 2020). In this study, both IRT and non-IRT data-generating processes were used to overcome this issue and these are described in detail in the upcoming subsections. The effect of varying test form difficulty difference, test taker ability, and the number of binary and polytomous items on each of the test forms was explored in different scenarios using both data-generating processes. A previous study has shown that IRTKE-equated scores are largely unaffected by sample size in a mixed-format setting (Wiberg & González, 2021). Consequently, we did not vary sample size and generated data from 1500 test takers in all simulated scenarios for both the IRT and non-IRT simulations.

### IRT Simulations

The GPC models fit to the SweSAT forms, summarized in Table 1, were used to simulate test scores in the IRT simulations. The model item parameters from the 2014 test form were used as a base scenario for all three test forms: X, Y, and A. The item parameters from X_SAT_ were used to generate both X and Y data. Note that this results in the true equating transformation *φ*(*x*) = *x*, because X and Y are the same test forms. The item parameters from the A_SAT_ GPC model fit using the 2014 test taker group were used to generate anchor test data.

We artificially constructed various test forms to vary the number of polytomous and dichotomous items in different scenarios. The polytomous items from the GPC model fit to the 2013 SweSAT test taker data were added to each test form to construct scenarios with a larger number of polytomous items. Dichotomous items were randomly removed to keep the same total scores on each test form.

To examine the effect of varying test form difficulty, we made the Y form easier in each scenario, while keeping the X and A forms unchanged. All items from the 2013 and 2014 SweSAT GPC models were placed into a large item pool. Then, items were randomly sampled from the easiest 67% (based on difficulty parameters) of this item pool until the scenario-specific number of polytomous and dichotomous items had been reached, thus creating an easier Y form.

Scenarios with equal and non-equal test taker populations were considered. In scenarios with equal test taker populations, test taker abilities were drawn from the standard normal distribution, N(0,1) , for both *P* and *Q*. In scenarios with non-equal test taker populations, the ability distribution for *P* was kept N(0,1) , while the distribution for *Q* was changed to N(0.5,1.2) . These parameter distributions have been used in previous studies and a population mean difference of .5 points is considered relatively large (Andersson, 2016; Meng, 2012; Ogasawara, 2003).

### Non-IRT Simulations

To generate data without using IRT models, we adopted an approach similar to Leôncio et al. (2022), generating total test scores directly from continuous probability distributions. To ensure realistic score distributions, probability density functions (pdfs) were fit to selected items from the SweSAT datasets using smoothing splines with the R package gss (Gu, 2014).

**Figure 2.**
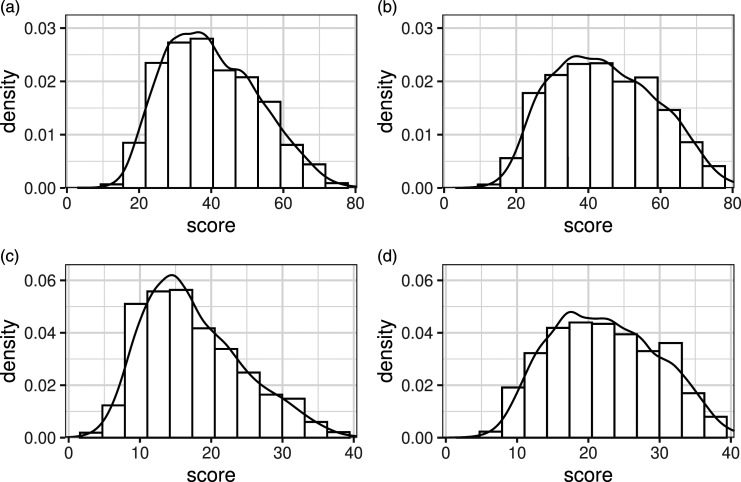
Spline-estimated pdfs used for simulation study data-generation. Each pdf is plotted on top of the relative score frequencies used to estimate the pdf. (a) X/Y (b) less difficult Y (c) A (d) more able group A.

The pdf in Figure 2(a) was fit to the scores on the X_SAT_ form and used to generate test scores on form X in all simulated scenarios. The continuous scores were rounded to obtain sum scores. To simulate scenarios with equal test form difficulty, the same pdf was used to generate form Y scores. To create scenarios in which the test forms differed in difficulty, a less difficult Y form was created. This was done by randomly sampling items from the 75% least difficult items (in the sense of percentage correct responses) on Y_SAT_ and A_SAT_ until a total score of 80 had been reached. The resulting pdf is shown in Figure 2(b).

To create scenarios in which the abilities of *P* and *Q* were equal, a pdf fit to A_SAT_ test scores from 2014 was used to generate anchor test scores for both test taker groups, see Figure 2(c). To create scenarios where the test taker populations differed in ability, a different anchor density function was created to generate anchor responses for the population taking the Y test. In a similar manner to how the more difficult Y test was created, items were sampled from the 75% least difficult ones on Y_SAT_ and A_SAT_ until a total test score of 40 had been reached. This results in an anchor test density function with higher probabilities for higher scores (reflecting more able students), see Figure 2(d). Note, however, that changing the distribution of the anchor test scores for one population indirectly alters the difficulty difference between X and Y, thus changing the true equating transformation. This is an undesirable consequence as we want to compare different scenarios varying one factor at a time. This problem was overcome by adjusting the distribution for *Y* in scenarios with non-equal anchor pdfs to match the test form difficulty differences in scenarios with equal anchor pdfs. The procedure is described in detail in Appendix B. Additionally, the scores for the anchor test need to be highly correlated with the scores for the main test forms in order for the anchor test to serve its intended purpose. In the simulations, these correlations were set to .85, which mimics the correlations found in the SweSAT forms, see Table 1. To achieve this, we used samples from bi-variate standard normal variables with the specified correlation using the mvrnorm() function in the MASS R package. The sampled values were then put through the standard normal cdf function to obtain their cumulative probabilities. Finally, the resulting probabilities were converted to spline distribution quantiles using the gss package.

Generating total test scores is sufficient to perform LLKE, but for IRTKE and IRTOSE, the scores for each item are required in order to fit the IRT models. To resolve this problem, an item response matrix of 1’s and 0’s was generated to match the simulated total scores. Note that randomly generating correct and incorrect responses, with equal probabilities for each item, until a total test score has been achieved would create an unrealistic situation as no item will appear to be particularly difficult or easy. Instead, item responses were generated using probability weights associated with each item. To mimic the true probabilities of getting each item correct in the SweSAT data, the weights were selected using an iterative procedure presented in Appendix D for each test form. The weights were computed for each scenario before running the actual simulations. To construct polytomous items, randomly chosen triplets of dichotomous items from the generated response matrix were summed together. The number of polytomous items was varied between 10, 15, and 20 for test forms X and Y, and between 5 and 10 for the anchor tests. They always contained four response categories, that is, a maximum score of 3.

### Performance Measures

Let ${\hat{\varphi }}_{l}\left(x\right)$ denote the estimated equating transformation from the *l*:th simulation iteration and $\bar{\varphi }\left(x\right)$ the mean of ${\hat{\varphi }}_{l}\left(x\right)$ across all 1000 iterations. In each simulated scenario, bias, SEE and root mean squared error (RMSE) were computed to evaluate equating performance locally at each score point on form X

$$\begin{array}{ll} \text{bias}\left(x\right) & =\bar{\varphi }\left(x\right)-\varphi \left(x\right)\\ \text{SEE}\left(x\right) & =\sqrt{\frac{1}{1000-1}\overset{1000}{\underset{l-1}{\sum }}{\left[{\hat{\varphi }}_{l}\left(x\right)-\bar{\varphi }\left(x\right)\right]}^{2}}\\ \text{RMSE}\left(x\right) & =\sqrt{\frac{1}{1000}\overset{1000}{\underset{l=1}{\sum }}{\left[{\hat{\varphi }}_{l}\left(x\right)-\varphi \left(x\right)\right]}^{2}}. \end{array}$$

We will refer to these as local measures. To measure overall performances, global measures were formed both by averaging each local measure over all X scores and by summing up and weighting each local measure by its X score prevalence. Specifically, the global measures considered were average absolute bias (AAB), average SEE (ASEE), average RMSE (ARMSE), weighted absolute bias (WAB), weighted SEE, and weighted RMSE (WRMSE).

The true equating transformation φ(x) must be known for estimation of bias and RMSE. Even using simulated data, what constitutes the true equating is not obvious. To circumvent this problem, we took the approach of defining one equating transformation as the true one (see e.g., Leôncio et al., 2022; Wang et al., 2008; Wiberg & González, 2016). For the non-IRT simulations, we defined φ(x) as the CE transformation $\varphi_{Y}\left(x\right)=F_{Y}^{-1}\left(F_{AQ}\left(F_{AP}^{-1}\left(F_{X}\left(x\right)\right)\right)\right)$ , computed using the spline fit cdfs. For the IRT simulations, φ(x) was defined as the IRTOSE transformation. See Appendix C for details. Using this equating method as the true equating transformation has been a common choice in simulation studies in which IRT models have been used to generate test data (e.g., Ogasawara, 2003; Wang et al., 2008).

### Simulation Results

Tables 2 and 3 show the global measures for the simulated scenarios containing 50 dichotomous and 10 polytomous items were used on test forms X and Y, together with 25 dichotomous and 5 polytomous items on the anchor test. With 1000 simulation iterations, the Monte Carlo SEs of the estimated performance measures (SEs of the simulation estimates) are smaller than .004 for all bias measures and smaller than .003 for all SEE measures. See Appendix E for formulas and derivation.

**Table 2.** Global Performance Measures From the Non-IRT Simulations. X ≠ Y Indicates Whether or Not the Test Forms Differed in Difficulty and *P ≠ Q* Indicates Whether or Not the Test Takers Taking Each Form Differed in Ability. The Bold Numbers Indicate the Smallest Value in Each Scenario.

Method	X ≠ Y	*P ≠ Q*	Non-IRT Simulation Results					
			AAB	WAB	ASEE	WSEE	RMSE	WRMSE
IRTOSE EG			.02	.02	.55	.56	.55	.56
IRTOSE PSE			.03	.03	.45	.44	.45	.44
IRTKE EG			**.01**	.02	.56	.57	.55	.57
IRTKE CE			.02	.02	**.38**	**.35**	**.38**	**.35**
IRTKE PSE			.02	.03	.45	.44	.45	.44
LLKE EG			.04	.02	.93	.64	.93	.64
LLKE CE			.02	.02	.63	.43	.63	.43
LLKE PSE			.02	**.01**	.59	.40	.59	.40
IRTOSE EG	x		.22	.12	.55	.62	.65	.64
IRTOSE PSE	x		.21	.12	.46	.50	.54	.52
IRTKE EG	x		.24	.12	.56	.62	.67	.64
IRTKE CE	x		.22	.09	**.38**	**.39**	**.49**	**.41**
IRTKE PSE	x		.24	.13	.47	.50	.57	.52
LLKE EG	x		**.14**	**.08**	.93	.69	.95	.70
LLKE CE	x		.32	.14	.61	.47	.72	.50
LLKE PSE	x		.30	.12	.58	.44	.68	.46
IRTOSE EG		x	6.19	8.37	.55	.58	6.22	8.39
IRTOSE PSE		x	1.40	1.62	.44	.47	1.52	1.72
IRTKE EG		x	6.22	8.37	.56	.58	6.25	8.39
IRTKE CE		x	.35	.20	**.39**	**.38**	**.56**	**.45**
IRTKE PSE		x	1.42	1.62	.45	.47	1.53	1.73
LLKE EG		x	6.32	8.35	.97	.65	6.50	8.38
LLKE CE		x	**.15**	**.08**	.71	.46	.72	.47
LLKE PSE		x	.92	1.27	.67	.43	1.27	1.36
IRTOSE EG	x	x	6.57	9.14	.56	.62	6.59	9.16
IRTOSE PSE	x	x	1.51	1.97	.45	.51	1.61	2.07
IRTKE EG	x	x	6.63	9.14	.57	.62	6.66	9.16
IRTKE CE	x	x	**.27**	.17	**.39**	**.41**	**.52**	**.47**
IRTKE PSE	x	x	1.55	1.98	.45	.51	1.65	2.07
LLKE CE	x	x	.45	**.15**	.70	.50	.87	.53
LLKE	x	x	6.66	9.13	.97	.69	6.82	9.16
LLKE PSE	x	x	1.34	1.41	.66	.47	1.51	1.50

**Table 3.** Global Performance Measures From the IRT Simulations. X ≠ Y Indicates Whether or Not the Test Forms Differed in Difficulty and *P ≠ Q* Indicates Whether or Not the Test Takers Taking Each Form Differed in Ability. The Bold Numbers Indicate the Smallest Value in Each Scenario.

Method	X ≠ Y	*P ≠ Q*	IRT Simulation Results					
			AAB	WAB	ASEE	WSEE	RMSE	WRMSE
IRTOSE EG			.04	.03	.52	.56	.53	.56
IRTOSE PSE			.03	.02	.43	.46	.44	.46
IRTKE EG			.03	.03	.52	.56	.53	.56
IRTKE CE			**.02**	**.01**	**.35**	**.32**	**.35**	**.33**
IRTKE PSE			**.02**	.02	.43	.46	.43	.46
LLKE EG			.04	.03	.80	.62	.80	.62
LLKE CE			.05	**.01**	.88	.47	.88	.47
LLKE PSE			.04	**.01**	.77	.43	.77	.43
IRTOSE EG	x		.04	**.01**	.52	.60	.54	.60
IRTOSE PSE	x		.04	.02	.46	.54	.48	.54
IRTKE EG	x		**.01**	.02	.53	.60	.53	.60
IRTKE CE	x		**.01**	**.01**	**.36**	**.36**	**.36**	**.36**
IRTKE PSE	x		.02	.02	.47	.54	.47	.54
LLKE EG	x		.18	.11	.78	.68	.83	.69
LLKE CE	x		.27	.11	.78	.50	.84	.52
LLKE PSE	x		.34	.15	.66	.45	.76	.48
IRTOSE EG		x	4.13	5.83	.52	.58	4.21	5.87
IRTOSE PSE		x	**.03**	**.04**	.43	.46	.43	.46
IRTKE EG		x	4.16	5.83	.53	.58	4.25	5.87
IRTKE CE		x	.22	.16	**.35**	**.33**	**.42**	**.38**
IRTKEPSE		x	**.03**	**.04**	.43	.46	.43	.46
LLKE EG		x	4.25	5.84	.77	.67	4.46	5.90
LLKE CE		x	.17	.17	.82	.48	.85	.52
LLKE PSE		x	.84	.87	.73	.46	1.22	1.03
IRTOSE EG	x	x	3.99	5.92	.55	.61	4.09	5.97
IRTOSE PSE	x	x	.05	**.04**	.44	.48	.45	.49
IRTKE EG	x	x	4.03	5.92	.56	.61	4.13	5.97
IRTKE CE	x	x	.21	.17	**.35**	**.35**	**.42**	**.40**
IRTKE PSE	x	x	**.03**	**.04**	.44	.48	.44	.49
LLKE EG	x	x	4.20	5.93	.79	.69	4.40	6.00
LLKE CE	x	x	.33	.22	.81	.53	.89	.58
LLKE PSE	x	x	.81	.88	.75	.51	1.21	1.06

When the test taker populations were identical, all methods performed relatively well in terms of AAB and WAB, see Tables 2 and 3. The results show only small differences between IRTKE and IRTOSE in all performance measures (comparing IRTKE EG to IRTOSE EG and IRTKE PSE to IRTOSE PSE). These differences are likely negligible in most practical settings. In the IRT simulations, the IRTKE methods exhibited lower bias compared to their LLKE counterparts in all scenarios. However, in the non-IRT simulations, there was no such clear distinction between the presmoothing methods. As expected, the EG methods were highly biased when the test taker populations differed. The CE methods outperformed the PSE method scenarios with different test taker populations in the non-IRT setting, while IRTKE PSE had the lowest AAB and WAB in all scenarios in the IRT simulations. The effect of changing test form difficulty was relatively small in terms of bias in comparison to the effect resulting from changing test taker ability. The WAB was generally smaller in scenarios where the test form difficulty was equal, provided all other factors remained the same.

IRTKE CE showed the smallest ASEE and WSEE in all scenarios in both the IRT and non-IRT settings. However, using CE resulted in larger ASEEs and WSEEs compared to PSE when LLKE was used. The effect on the SEEs from changing test difficulty and population ability was relatively small. In the non-IRT simulations, the use of the anchor test using the NEAT design resulted in lower ASEE and WSEE compared to the EG methods even in scenarios where populations were the same, see Table 2. As shown in Table 3, similar findings were observed in the IRT simulations, although LLKE EG showed lower ASEE than LLKE CE in three out of four scenarios. In terms of ARMSE and WRMSE, IRTKE CE performed the best among all methods in both the IRT and non-IRT simulations.

**Figure 3.**
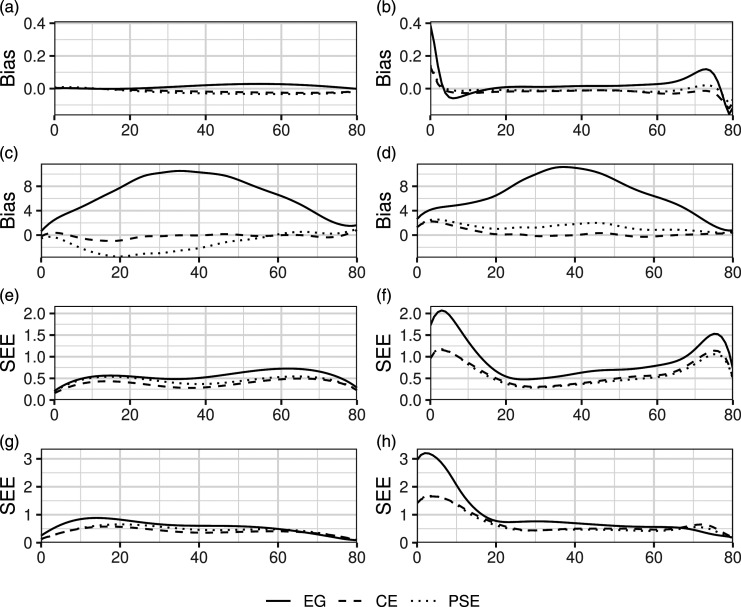
Estimated bias and SEE in selected scenarios from the non-IRT simulations. The *x*-axis shows the test score of form X. *X* ≠ *Y* indicates whether or not the test forms differed in difficulty and *P* ≠ *Q* indicates whether or not the test takers taking each form differed in ability. (a) IRTKE X = Y *P = Q* (b) LLKE X = Y *P = Q* (c) IRTKE X ≠ Y *P ≠ Q* (d) LLKE X ≠ Y *P ≠ Q* (e) IRTKE X = Y *P = Q* (f) LLKE X = Y *P = Q* (g) IRTKE X ≠ Y *P ≠ Q* (h) LLKE X ≠ Y *P ≠ Q*.

Figure 3 presents plots of the simulation estimated bias and SEE for each method in the non-IRT simulations. Plots for IRTOSE were omitted due to its close similarities with IRTKE. The plotted curves correspond to scenarios with equal test form difficulty, 50 dichotomous and 10 polytomous items on test forms X and Y along with 25 dichotomous and 5 polytomous items on the anchor test. As shown in Figure 3(a), the IRTKE methods were largely unbiased when the populations and test forms were equal, while LLKE showed some bias at the edges of the score scale, see Figure 3(b). When comparing Figure 3(a) and (b) with Figure 3(c) and (d), it is clear that population differences together with differences in test form difficulty led to increased bias and enlarged the differences in bias between the equating methods. As illustrated in Figure 3(e)–(h), the SEEs were larger at the upper and lower ends of the score scale for the LLKE methods when compared to their IRTKE counterparts in all scenarios. These findings were consistent throughout all simulated scenarios using both data-generating processes. For plotted curves, see Appendix F.

No effects on bias, SEE, or RMSE from varying the number of polytomous and dichotomous items in the main and/or anchor tests could be identified. Consequently, results from scenarios with numbers of dichotomous/polytomous items differing from those already presented have been omitted, but can be obtained upon request from the corresponding author.

## Discussion

In this study, the efficiency of IRTKE for mixed-format tests was evaluated. IRTKE was contrasted against its close methodological relatives, IRTOSE and LLKE, under both EG and NEAT data collection designs. In a real data setting, we used data from two test forms from the Swedish national test in mathematics and two test forms from a college admission test. In a simulation study, we further evaluated the equating performance when different conditions were varied, including the ability of the test taker populations, test form difficulty, and the proportions of dichotomously and polytomously scored items in the test forms.

In all comparisons, IRTKE consistently provided similar equated scores and SEEs as IRTOSE. This indicates that the choice of presmoothing method has a much larger effect on the equated scores compared to whether or not one uses equipercentile equating or kernel equating to smooth out the score distributions. Despite the similarities in the equated scores, IRTKE could potentially be seen as a more attractive method, as the kernel equating framework allows for analytically computed SEEs. Kernel equating also has intuitive appeal in that the smoothed score distribution retains the same mean and standard deviation as the discrete scores.

IRT-presmoothing models were found to provide smaller SEEs at the lower and upper ends of the score scales when compared to log-linear models. This property of IRTKE/IRTOSE was consistent throughout the real data examples and all simulated scenarios. Similar findings were also observed in the simulation study conducted by Andersson and Wiberg (2017), where IRTKE and LLKE were compared using dichotomous items. A possible explanation for this phenomenon is that log-linear models, as opposed to IRT models, directly smooth the observed score frequencies of each total test score. As a result, a few extra test takers in the lower and/or upper ends of the test score scale due to sampling error typically leads to a shift in the lower and/or upper quantiles of the presmoothed distribution when log-linear models are used. In contrast, with IRT presmoothing, item response curves are fit for each item, and the item response curves (which are typically monotone) of the chosen IRT model are enforced upon all items. In other words, smoothing is done at an item level, and the parametric form of the chosen IRT model lowers the effect of random “bumps” in the total score sample frequencies. As illustrated in this study and the study by Andersson and Wiberg (2017), IRT presmoothing is an effective way of reducing the SEEs at the lower and upper ends of the score scale where there are typically fewer test takers in the data. One should note that small SEEs do not necessarily indicate good equating, and the IRT approach could potentially introduce bias at the extremes if the chosen IRT model is a poor fit. Since no true equating transformation is known in a practical setting, multiple equating methods should be considered for comparison purposes. If no method appears to be clearly inaccurate, and if there is no reason to prefer one over another, a possible solution could be to use the average results from different methods (Holland & Strawderman, 2011).

From the simulations, it is clear that ability differences between the test taker populations led to relatively large bias for LLKE PSE compared to its CE alternative. This is also in line with previous research in non–mixed-format settings (Mao et al., 2006; Wang et al., 2008). Wang et al. (2008) recommended the use of PSE in situations where the population differences are small, because of smaller SEEs obtained using the PSE method when compared to the values obtained with CE. However, in our study, similar findings were only observed when using log-linear presmoothing models. When IRTKE was used, CE showed smaller average SEEs compared to PSE in both the real-data example and the simulation study. The smaller SEEs led to smaller RMSEs even in scenarios where IRTKE PSE produced less biased results. For this reason, we recommend CE over PSE when conducting equatings under the NEAT design using the IRTKE framework. It should be noted that these results only apply when using the mean-mean IRT coefficient linking method for IRTKE PSE, as this was the only approach considered in this study. Previous studies on coefficient linking methods have shown that the Haebara and Stocking–Lord methods tend to outperform moment-based methods such as the mean-mean method (e.g., Andersson, 2018). However, to what extent the linking method affects the actual IRTKE PSE equatings remains unclear, and is a topic for future study.

In our simulation study, we compared the results from two types of data-generating processes. Both were based upon real test data from the SweSAT, but used different true equating transformations for bias estimation. In the non-IRT simulations, where the true equating transformation was defined using CE, the CE methods had smaller bias than the PSE methods. In contrast, in the IRT simulations, where the IRT observed score equating transformation for a synthetic population was defined as the true equating transformation, IRTKE PSE had the lowest bias. As expected, the bias was generally smaller when IRTKE was used in the IRT simulations, while the results differed between scenarios in the non-IRT simulations. The contradicting results between the IRT and the non-IRT simulations highlight the importance of selecting appropriate data-generating processes and true equating transformations when conducting simulation studies. This issue has been previously discussed in the study by Wiberg and González (2016), where the authors encouraged the use of multiple true equating transformations for more fair comparisons between methods. In our study, we used two different processes for generating test data, each with their own true equating. If we had only used IRT simulations, which is the more common approach, it would have appeared that IRTKE PSE outperformed the other methods in terms of bias. We argue that these types of studies where multiple simulation methods are used result in a more fair comparison, even though in this case the results are less conclusive.

One limitation of the current study is that strictly unidimensional test forms were assumed throughout the simulations. However, in a multidimensional setting, when item types measure different constructs, as is common in many practical situations (Lee & Lee, 2014; Tate, 2000; Wang & Kolen, 2014), there would probably be a larger impact resulting from varying the number of items of each item type. This should be investigated further.

Another limitation is that we used only GPC models to model both dichotomous and polytomous items during IRTKE. As the sample sizes are often large in situations where equating is conducted, it would be of interest to explore the effects on the estimated equating transformations of using simultaneous calibration of mixed IRT models (Chon et al., 2010). For example, a three parameter logistic model for dichotomous items (which are commonly multiple-choice items) and a GPC model for polytomous items could be used. In this way, the effect of guessing on the dichotomous items would be modelled without increasing the model complexity for polytomous items. Additionally, parametric IRT models sometimes do not fit the data, and the possibility of using non-parametric models (e.g., Arenson & Karabatsos, 2018; Wiberg et al., 2019) in the presmoothing step should also be explored.

In conclusion, when compared to LLKE and IRTOSE, IRTKE appears to be a promising approach for mixed-format test data. IRTKE outperformed LLKE in terms of RMSE and SEE in most simulated scenarios, with a larger difference for both high and low scoring test takers. In both the simulations and the empirical study, IRTKE and IRTOSE produced similar results. However, a key distinguishing factor is that IRTKE leverages several advantages inherent to kernel equating. For example, it facilitates analytical SEEs and provides considerable flexibility in the continuization of discrete score distributions through adjustable bandwidth. These attributes underscore the potential of IRTKE as a potent tool in test equating methodologies.

## Supplemental Material

Supplemental Material - Efficiency Analysis of Item Response Theory Kernel Equating for Mixed-Format Tests Click here for additional data file. Supplemental Material for Efficiency Analysis of Item Response Theory Kernel Equating for Mixed-Format Tests by Joakim Wallmark, Maria Josefsson, and Marie Wiberg in Applied Psychological Measurement.
