# Supplementary material for: Efficiency Analysis of Item Response Theory Kernel Equating for Mixed-Format Tests
Source: Appl Psychol Meas. 2023 Oct 19;47(7-8):496–512. doi: 10.1177/01466216231209757 (PMC10664743; doi:10.1177/01466216231209757)
Supplement: Supplemental Material - Efficiency Analysis of Item Response Theory Kernel Equating for Mixed-Format Tests [file sj-pdf-1-apm-10.1177_01466216231209757.pdf]

A Bandwidths

Table A1: Kernel equating bandwidths selected by penalty function minimization.

**Kernel equating bandwidths**				
Method	h_X	h_Y	h_{AP}	h_{AQ}
IRTKE EG	0.59	0.62	-	-
LLKE EG	0.62	0.63	-	-
IRTKE CE	0.68	0.68	0.63	0.63
LLKE CE	0.65	0.66	0.59	0.59
IRTKE PSE	0.65	0.66	-	-
LLKE PSE	0.69	0.68	-	-

B Finding the test score distribution for test form Y for non-IRT simulations when populations differ

Assume a simulation scenario (S1) in which $F_X(x)$ and $F_Y(y)$ are known and the abilities of test taker populations P and Q are equal. In this scenario, the true equating transformation is $\varphi(x) = F_Y^{-1}(F_X(x))$. We aim to simulate another scenario (S2) where a difference in abilities between P and Q is introduced, without altering the differences in test form difficulty. Assuming $F_{AP}(a)$ and $F_{AQ}(a)$ are known, we want to find a new score distribution for form Y, $F_{Y2}(y)$, such that $\varphi(x)$ in S2 is the same as in S1. Assuming the CE transformation is the true one we get

$$
\begin{aligned}
\varphi(x) &= F_{Y2}^{-1}(F_{AQ}(F_{AP}^{-1}(F_X(x)))) \\
F_{Y2}(\varphi(x)) &= F_{AQ}(F_{AP}^{-1}(F_X(x))) \\
F_{Y2}(y) &= F_{AQ}(F_{AP}^{-1}(F_X(\varphi^{-1}(y)))) \\
F_{Y2}(y) &= F_{AQ}(F_{AP}^{-1}(F_X(F_X^{-1}(F_Y(y))))) \\
F_{Y2}(y) &= F_{AQ}(F_{AP}^{-1}(F_Y(y)))
\end{aligned}
$$

It is evident that the CDF of $F_{Y2}(y)$ is a function of $F_{AQ}(a)$, $F_{AP}(a)$ and $F_Y(y)$. Its inverse $F_{Y2}^{-1}(\cdot) = F_Y^{-1}(F_{AP}(F_{AQ}^{-1}(\cdot)))$ can be used to generate Y scores corresponding to the same difficulty difference between forms X and Y as when using $F_X(x)$ and $F_Y(y)$ and equal populations.

C True equating transformation, IRT simulations

Algorithm C1 True equating transformation for IRT simulated data

1: Generate a sequence of ability values, Θ_P, evenly distributed over the most plausible range of abilities for population P.
2: For each ability value, obtain the score probabilities for each item using the GPC model with the true IRT parameters from the scenario of interest.
3: For each ability value and the probabilities from previous step, calculate the sum score probabilities $P_P(X=x|\theta)$ for each possible x using the recursive algorithm described in Thissen et al. (1995).
4: Let $f_{\theta_P}(\theta)$ be the ability density function for population P. Approximate the integral $P_P(X=x) = \int P_P(X=x|\theta) f_{\theta_P}(\theta) d\theta$ by summing over all ability values in Θ_P and weighting each conditional probability from the previous step with the relative likelihood of each θ

$$\hat{P}_P(X=x) = \sum_{u \in \Theta_P} \frac{P_P(X=x|\theta=u) f_{\theta_P}(u)}{\sum_{v \in \Theta_P} f_{\theta_P}(v)}$$

5: Repeat steps 1 to 4 for population Q.
6: Obtain the sum score probabilities for the target population, $T = 0.5P + 0.5Q$, using a weighted average of the results from each population $\hat{P}_T(X=x) = 0.5\hat{P}_P(X=x) + 0.5\hat{P}_Q(X=x)$.
7: Repeat step 2 to 6 for test form Y.
8: Perform equipercentile equating using the probabilities obtained from previous steps. The **equate** R package was used for this step.

D Algorithm for item weight selection

Algorithm D1 Selecting realistic item probability weights for simulations

1: Compute the probability of responding correctly to each SweSAT item used to fit the smoothing spline density function for the test form of interest (the ratio between the number of correct responses and the total number of responses for each item). The goal is to find weights which make simulated item score probabilities mimic these probabilities.
2: Generate a large amount of total test scores using the fitted spline density function. We used 100,000. A larger number results in more accurate performance estimates of the weights.
3: Initially set the weight for each item to 0.5 and set the initial value of the step parameter S, used later in the algorithm to adjust the weights, to 0.3. A larger item weight implies an item is easier, and more likely to be scored as correct.
4: Generate item responses using the weights from step 3 for all total scores from step 2. Items with correct responses were sampled using the **sample** function in R to which the weights can be supplied through the **prob** argument.
5: Calculate the probability for getting each item correct in the large dataset of 100,000 test takers generated using the weights in step 4. The ratio between the number of correct responses and the total number of responses for each item were used for the calculations.
6: For each item, compare the probability of getting the item correct (step 1) with the item correct probability received using the weights (previous step). If the probability from using the current weight is too low, increase the weight with the current value of S. If it is too high, decrease the weight with S.
7: Decrease the value of S with 25%.
8: Repeat steps 4-6 until $S < 0.01$.

E Monte Carlo standard errors

In this section, we derive the formulas to calculate Monte Carlo SEs for AAB, WAB, ASEE and WSEE. We use the notation Bias_x and SEE_x to denote the estimated bias and SEE for a total score of x on the test form from which we equate from. From Table 6 of Morris et al. (2019), we have, for 1000 simulation iterations, variances

$$\begin{aligned}\text{Var}(\text{Bias}_x) =& \frac{\text{SEE}_x^2}{1000}\\ \text{Var}(\text{SEE}_x) =& \frac{\text{SEE}_x^2}{2(1000-1)}\end{aligned}$$

which gives, for a weighted sum of 81 test scores (0, 1, ..., 80)

$$\begin{aligned}\text{Var}(\text{WAB}) =& \text{Var}(\sum_{i=0}^{80} w_i \text{Bias}_i) = \sum_{i=0}^{80} w_i^2 \text{Var}(\text{Bias}_i) = \frac{1}{1000} \sum_{i=0}^{80} w_i^2 \text{SEE}_i^2\\ \text{Var}(\text{WSEE}) =& \text{Var}(\sum_{i=0}^{80} w_i \text{SEE}_i) = \sum_{i=0}^{80} w_i^2 \text{Var}(\text{SEE}_i) = \frac{1}{2(1000-1)} \sum_{i=0}^{80} w_i^2 \text{SEE}_i^2\\ \text{SE}(\text{WAB}) =& \sqrt{\text{Var}(\text{WAB})}\\ \text{SE}(\text{WSEE}) =& \sqrt{\text{Var}(\text{WSEE})}\end{aligned}$$

where w_i is the weight given to item i. For AAB and ASEE, we use the above formulas with $w_i = 1/81$ for all items.

F Simulation results

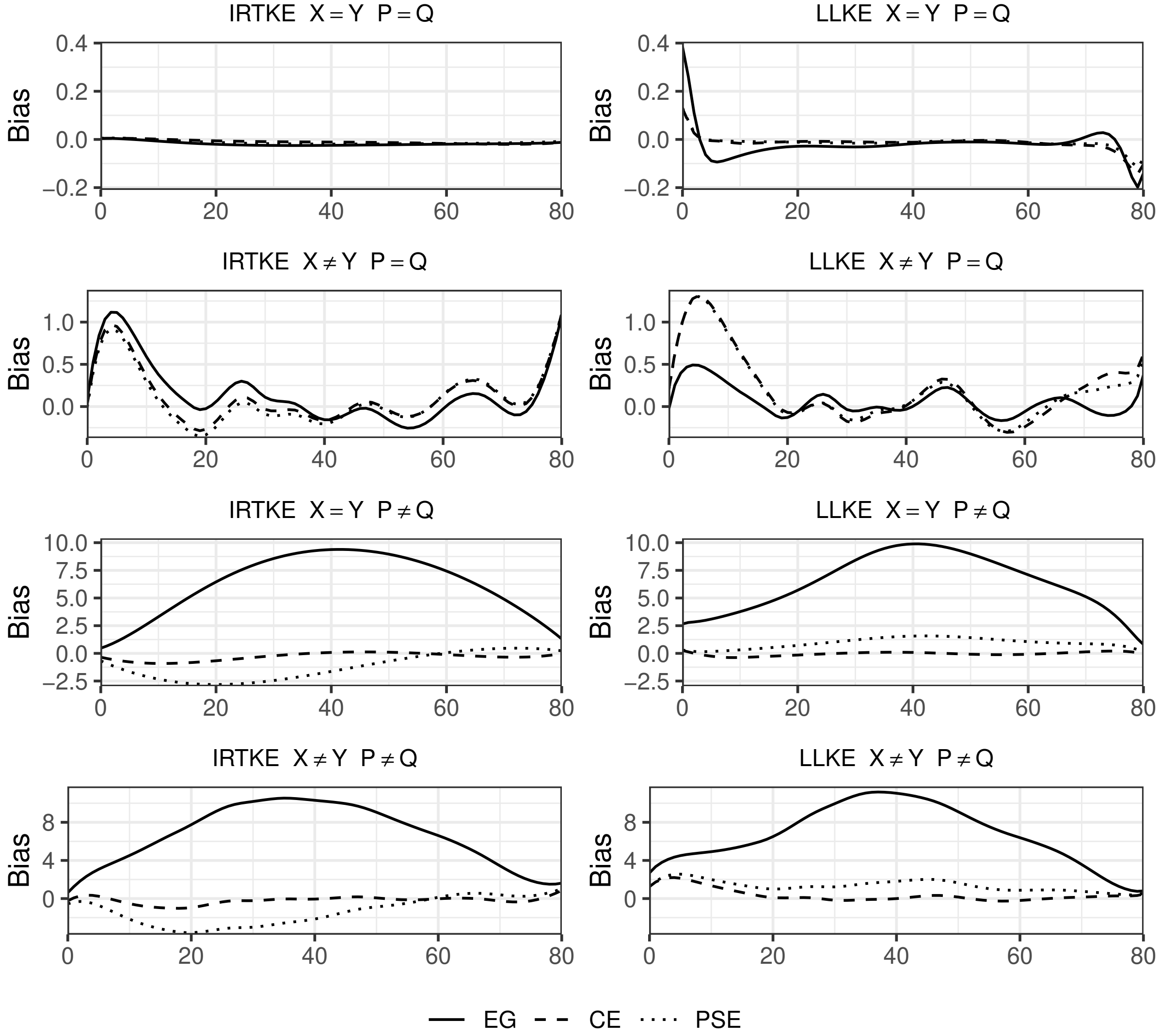

Figure F1: Bias plots from the non-IRT simulations. The x-axis shows the test score of form X. $X \neq Y$ indicates whether or not the test forms differed in difficulty and $P \neq Q$ indicates whether or not the test takers taking each form differed in ability.

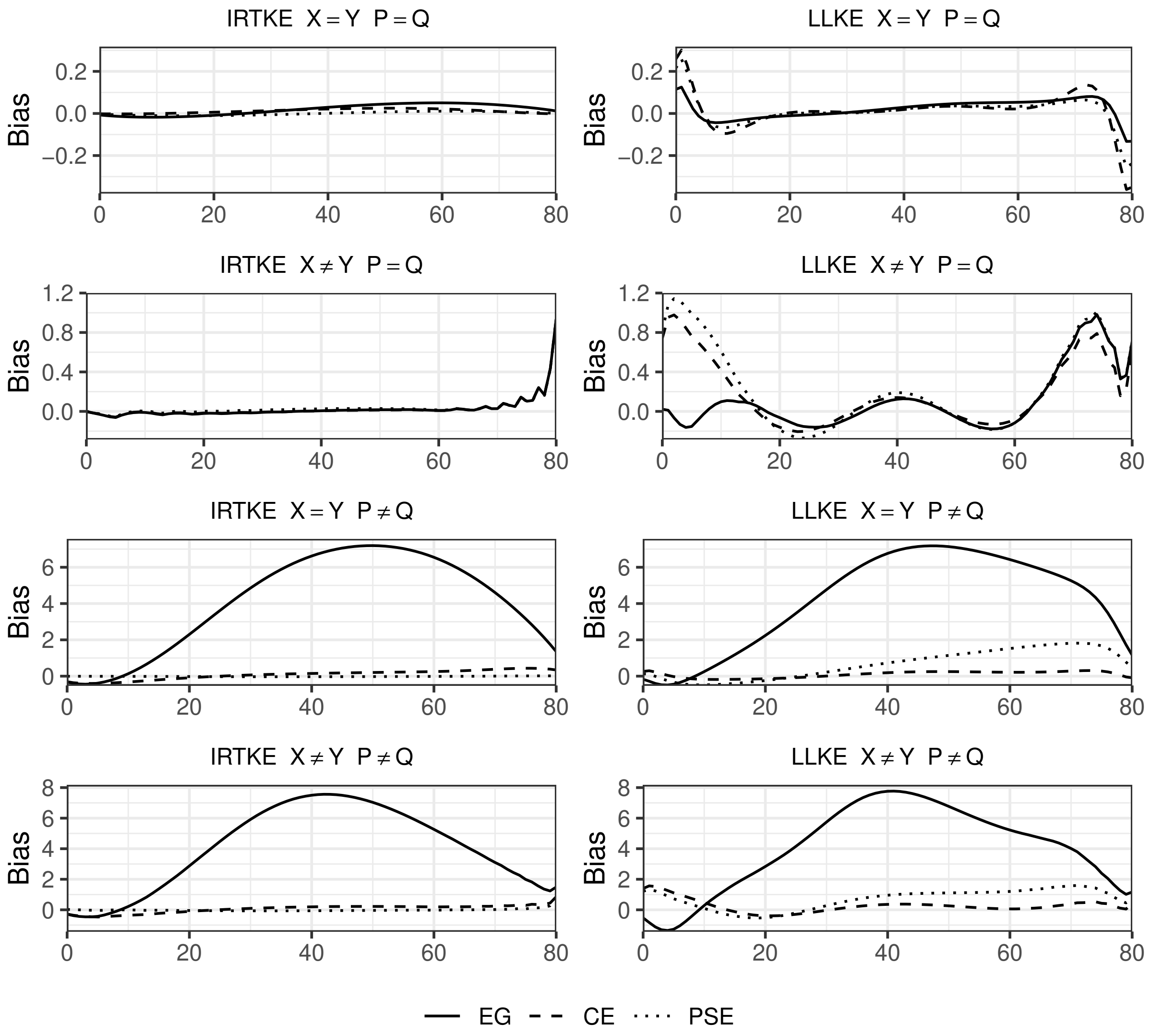

Figure F2: Bias plots from the IRT simulations. The x-axis shows the test score of form X. $X \neq Y$ indicates whether or not the test forms differed in difficulty and $P \neq Q$ indicates whether or not the test takers taking each form differed in ability.

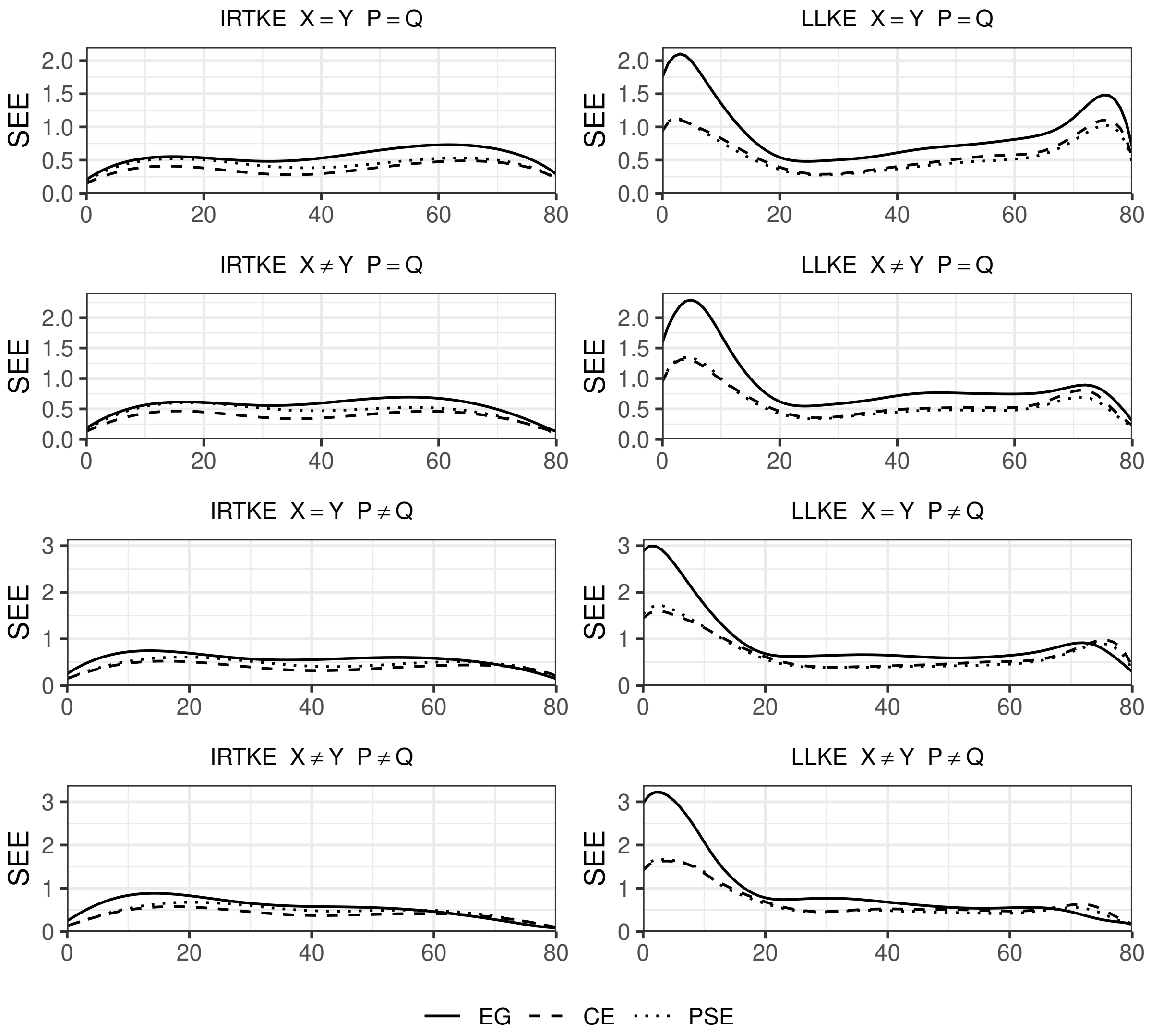

Figure F3: SEE plots from the non-IRT simulations. The x-axis shows the test score of form X. $X \neq Y$ indicates whether or not the test forms differed in difficulty and $P \neq Q$ indicates whether or not the test takers taking each form differed in ability.

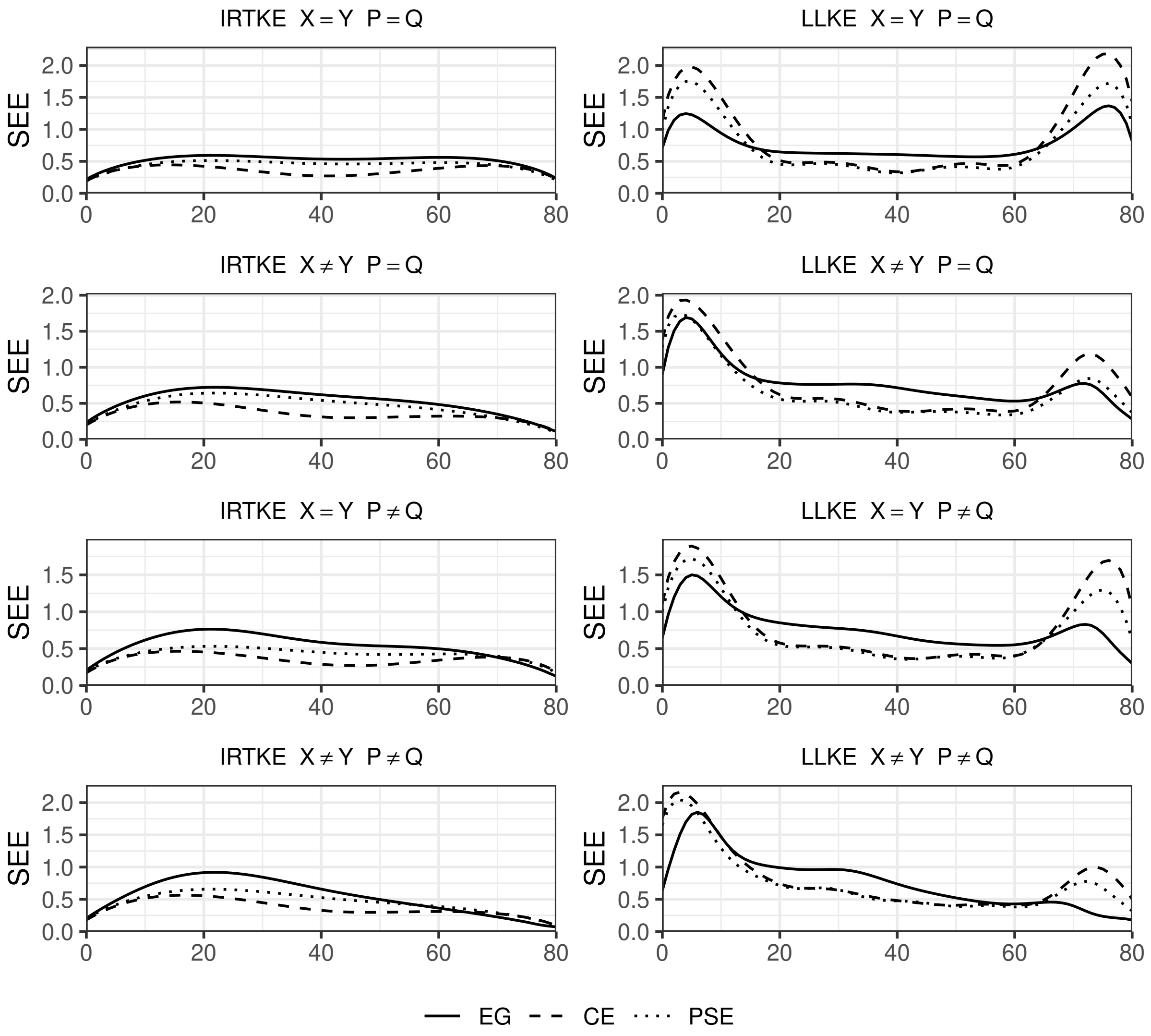

Figure F4: SEE plots from the IRT simulations. The x-axis shows the test score of form X. $X \neq Y$ indicates whether or not the test forms differed in difficulty and $P \neq Q$ indicates whether or not the test takers taking each form differed in ability.

References

Morris, T. P., I. R. White, and M. J. Crowther (2019). Using simulation studies to evaluate statistical methods. *Statistics in Medicine 38*(11), 2074–2102.

Thissen, D., M. Pommerich, K. Billeaud, and V. S. L. Williams (1995). Item response theory for scores on tests including polytomous items with ordered responses. *Applied Psychological Measurement 19*(1), 39–49.
